# Cross-Variant Neutralizing Serum Activity after SARS-CoV-2 Breakthrough Infections

**DOI:** 10.3201/eid2805.220271

**Published:** 2022-05

**Authors:** Pinkus Tober-Lau, Henning Gruell, Kanika Vanshylla, Willi M. Koch, David Hillus, Philipp Schommers, Isabelle Suárez, Norbert Suttorp, Leif Erik Sander, Florian Klein, Florian Kurth

**Affiliations:** Charité–Universitätsmedizin Berlin, Berlin, Germany (P. Tober-Lau, W.M. Koch, D. Hillus, N. Suttorp, L.E. Sander, F. Kurth);; University of Cologne, Cologne, Germany (H. Gruell, K. Vanshylla, P. Schommers, I. Suárez, F. Klein);; German Center for Infection Research, Bonn-Cologne, Germany (F. Klein);; Bernhard Nocht Institute for Tropical Medicine, Hamburg, Germany (F. Kurth);; University Medical Centre Hamburg-Eppendorf, 20359, Hamburg (F. Kurth)

**Keywords:** COVID-19, 2019 novel coronavirus disease, coronavirus disease, severe acute respiratory syndrome coronavirus 2, SARS-CoV-2, viruses, respiratory infections, zoonoses, vaccines, mRNA, immunity, B cell, neutralizing antibody

## Abstract

To determine neutralizing activity against the severe acute respiratory syndrome coronavirus 2 ancestral strain and 4 variants of concern, we tested serum from 30 persons with breakthrough infection after 2-dose vaccination. Cross-variant neutralizing activity was comparable to that after 3-dose vaccination. Shorter intervals between vaccination and breakthrough infection correlated with lower neutralizing titers.

The B.1.1.529 (Omicron) variant of concern of severe acute respiratory syndrome coronavirus 2 (SARS-CoV-2) carries a high number of nonsynonymous mutations in the spike glycoprotein, relative to that of the ancestral (wild-type) strain (Wu01). Those mutations result in a strong immune evasion phenotype, as demonstrated by severely reduced serum neutralization after vaccination or previous infection with ancestral variants in most persons (*1*–*3*), lower vaccine effectiveness, and increased rates of reinfection (N. Andrews et al., unpub. data, https://www.medrxiv.org/content/10.1101/2021.12.14.21267615v1). However, booster vaccinations with 1 dose of mRNA vaccine after priming with an initial 2 doses induce high levels of serum neutralizing activity against Omicron (*1*,*4*). Substantial efforts have therefore been made to speed up booster vaccination campaigns in light of the rapid spread of Omicron and the recent surge of infections worldwide. Breakthrough infections after 2-dose mRNA vaccination can result in a natural boost to humoral immunity against SARS-CoV-2 (*5*; L.J. Abu-Raddad et al., unpub. data, https://www.medrxiv.org/content/10.1101/2022.01.18.22269452v2), and emerging evidence suggests that breakthrough infections with non-Omicron SARS-CoV-2 variants also elicit cross-neutralizing serum activity against Omicron (*6*). 

We determined serum neutralizing activity against the spike pseudotypes of SARS-CoV-2 Wu01 strain and 4 variants of concern (Alpha, Beta, Delta, Omicron [BA.1]) in 20 persons with non-Omicron (Alpha, Delta) SARS-CoV-2 infection after 2-dose mRNA vaccination with BNT162b2 (Comirnaty; Pfizer-BioNTech, https://www.comirnaty.com) or heterologous vaccination with ChAdOx1 (Vaxzevria; AstraZeneca, https://www.astrazeneca.com) and BNT162b2 (Appendix). We compared serum neutralization activity for this cohort with that of 2 age-matched cohorts, 1 consisting of 20 persons who received 2 or 3 doses of mRNA vaccine (*1*) and did not experience breakthrough infection and another cohort of 10 persons who experienced Omicron breakthrough infection after 2-dose vaccination (Figure, panel A; Appendix Table).

We detected significantly higher serum neutralizing activity against all investigated variants in serum from vaccinated persons with subsequent non-Omicron SARS-CoV-2 infection (Figure, panel B) than in serum from persons who received the regular 2 doses of vaccine and experienced no subsequent infection. The geometric mean 50% inhibitory serum dilution (ID_50_) against Wu01 was 6.3-fold higher after breakthrough infection (640 [95% CI 409–1,003] vs. 4,056 [95% CI 2,174–7,568]). This difference in serum neutralizing activity was particularly pronounced against the Beta (23.5-fold higher ID_50_, 49 [95% CI 28–85] vs. 1,148 [95% CI 524–2,514]) and Omicron (23.8-fold higher ID_50_, 9 [95% CI 5–13] vs. 202 [95% CI 79–515]) variants, each of which exhibits substantial immune escape. The boosting effect of non-Omicron breakthrough infections was highly variable (Figure, panel B) because serum neutralizing titers (ID_50_) showed a strong correlation with the interval between second vaccination and diagnosis of breakthrough infection (Omicron, Spearman ρ = 0.8299, p<0.0001; Wu01, ρ = 0.7048, p = 0.0005) (Figure, panel C; Appendix Figure, panels A–C). Breakthrough infections acquired >3 months after the second vaccination resulted in serum neutralizing capacity against both Wu01 and Omicron, which was comparable to that after 3-dose vaccination. This effect was observed after both non-Omicron and Omicron breakthrough infections (Figure, panel D). Similarly, neutralizing capacity against the Delta variant was increased after Omicron breakthrough infections (Appendix Figure, panel D). Limitations of this study include limited sample size and application of a pseudovirus-based neutralization assay.

**Figure:**
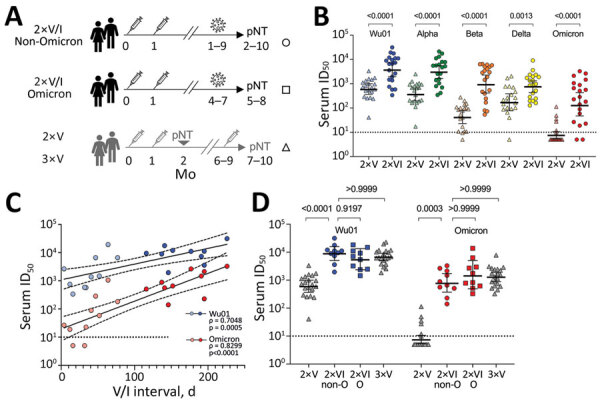
SARS-CoV-2 serum neutralizing titers across variants after postvaccination breakthrough infection. A) Schematic of the study cohort of 2×VI patients and age-matched reference cohorts (*1*). B) Serum neutralizing activity against Wu01 and SARS-CoV-2 variants in 2×V persons (triangles) and 2×V/I persons (circles). Horizontal lines indicate geometric mean ID_50_s; error bars, 95% CIs. Groups were compared by using the Mann-Whitney test. p values are shown at top. C) Correlation of serum neutralizing activity against SARS-CoV-2 Wu01 (blue) or Omicron (red) and interval between second vaccination and non-Omicron breakthrough infection (Spearman ρ and p values). Breakthrough infections within 3 months (90 days) from vaccination are indicated by light shaded symbols. Solid lines indicate linear regression, and dashed lines indicate 95% CIs. Correlation was determined by Spearman ρ. D) Serum neutralizing activity against SARS-CoV-2 Wu01 (blue) and Omicron (red) in 2×V or 3×V persons (triangles) compared with 2×V/I non-Omicron (circles) or Omicron (triangles) persons after 2 and 3 doses of mRNA vaccine. Only persons with vaccine-to-infection intervals >3 months are shown. Groups were compared by using the Kruskal-Wallis test with the Dunn multiple testing correction. Horizontal lines indicate geometric mean ID_50_s; error bars, 95% CIs. p values are shown at top. Black dotted lines in panels B, C, and D indicate the lower limit of quantification (ID_50_ = 10); ID_50_s <10 were imputed to half the lower limit of quantification (ID_50_ = 5). ID_50_, 50% inhibitory serum dilution; O, Omicron; pNT, pseudovirus neutralization test; SARS-CoV-2, severe acute respiratory syndrome coronavirus 2; V/I, vaccination with subsequent breakthrough infection; Wu01, ancestral (wild-type) SARS-CoV-2 strain; 2xV/I non-Omicron, vaccinated persons with non-Omicron breakthrough infection that occurred 1–8 months after vaccination (circles); 2xV/I Omicron, vaccinated persons with Omicron breakthrough infection that occurred 4–7 months after vaccination (squares); 2xV, vaccinated persons after 2 doses of mRNA vaccine; 3xV, vaccinated persons after 3 doses of mRNA vaccine (triangles).

In summary, we found that Omicron and non-Omicron SARS-CoV-2 breakthrough infections elicit cross-variant neutralizing antibodies. Our results suggest that short vaccination-to-infection intervals correlate with lower neutralizing titers, which may be relevant for recommendations concerning additional booster vaccination of persons who experience early breakthrough infections after initial immunization.

Appendix Additional methods for study of cross-variant neutralizing antibodies after SARS-CoV-2 breakthrough infections.
