# Supplementary material for: Cross-Variant Neutralizing Serum Activity after SARS-CoV-2 Breakthrough Infections
Source: Emerg Infect Dis. 2022 May;28(5):1050–2. doi: 10.3201/eid2805.220271 (PMC9045428; doi:10.3201/eid2805.220271)
Supplement: Appendix — Additional methods for study of cross-variant neutralizing antibodies after SARS-CoV-2 breakthrough infections. [file 22-0271-Techapp-s1.pdf]

Article DOI: https://doi.org/10.3201/eid2805.220271

Cross-Variant Neutralizing Serum Activity after SARS-CoV-2 Breakthrough Infections

Appendix

Patients, Materials and Methods

Ethics Statement

Subjects were recruited under protocols approved by the ethics committee (EC) of Charité-Universitätsmedizin Berlin (PaCOVID-19 (*1*) Study, EA2/066/20, DRKS00021688) or the EC of the Medical Faculty of Cologne (20–1187), conducted in accordance with the Declaration of Helsinki and Good Clinical Practice principles (ICH 1996). Written informed consent was obtained from all patients or legal representatives according to regulations set by the ethics committees of Charité - Universitätsmedizin Berlin and the Medical Faculty of Cologne, respectively.

Clinical data

Study participants were eligible for inclusion in case of PCR-confirmed SARS-CoV-2 infection following vaccination with 2 doses of BNT162b2, 2 doses of mRNA-1273, or 1 dose of ChAdOx followed by 1 dose of BNT162b2 ("breakthrough cases"). Individuals with non-Omicron breakthrough infections were diagnosed between February and November 2021, before the emergence of SARS-CoV-2 Omicron variant (B.1.1.529). Out of 20 individuals, seven were diagnosed with Alpha (B.1.1.7), nine with Delta (B.1.617.2). In four cases sequencing data was not available. Individuals with Omicron breakthrough infections were diagnosed in December 2021. Two out of 20 (10%) patients with non-Omicron and 1 out of 10 (10%) with Omicron SARS-CoV-2 infection were asymptomatic, the remaining patients exhibiting mild symptoms. Non-infected vaccinated individuals were recruited from the previously published prospective observational cohort studies EICOV, COVIMMUNIZE, and COVIM, approved by the EC of Charité - Universitätsmedizin Berlin (EA4/245/20 and EA4/244/20), by the Federal Institute for Vaccines and Biomedicines (Paul Ehrlich Institute), and the EC of the state of Berlin (*2–4*). To

detect concurrent SARS-CoV-2 infection, all participants received nucleic acid amplification tests at time of sampling, and all samples were tested for anti-nucleocapsid antibodies using the SeraSpot Anti-SARS-CoV-2 IgG microarray-based immunoassay (Seramun Diagnostica). Participants with history of infection, determined by self-reporting, positive nucleic acid amplification test, or presence of nucleocapsid antibodies according to the manufacturer's specifications were excluded from analysis. Further cohort details are described in the Appendix Table. Serum samples were stored at −80°C. All study participants provided written informed consent.

Pseudovirus neutralisation assays

Serum neutralising activity was determined using a single-round infection lentivirus-based assay (*5*,*6*). Pseudoviruses were generated in HEK293T cells by co-transfecting plasmids encoding the SARS-CoV-2 spike protein, HIV-1 Tat, HIV-1 Gag/Pol, HIV-1 Rev, and luciferase using the FuGENE 6 Transfection Reagent (Promega). Culture supernatant was replaced after 24 hours, and pseudovirus-containing supernatants were harvested at 48 h to 72 h after transfection. After centrifugation and filtration (0.45 μm), pseudoviruses were stored at −80°C until use. Pseudovirus titers were determined by infecting 293T-ACE2 cells and luciferase activity was measured in relative light units (RLUs) following a 48-hour incubation period at 37°C and 5% CO2 using a microplate reader (Berthold), by adding luciferin/lysis buffer (10 mM MgCl2, 0.3 mM ATP, 0.5 mM Coenzyme A, 17 mM IGEPAL (all Sigma-Aldrich), and 1 mM D-Luciferin (GoldBio) in Tris-HCL). Serum was heat-inactivated at 56°C for 45 min before use. Serial serum dilutions (1:3 dilution series starting at 1:10) were co-incubated with pseudovirus supernatants for 1 hour at 37°C before addition of 293T-ACE2 cells. After 48-hours at 37°C and 5% CO2, luciferase activity was measured as described above. After subtracting background RLUs of non-infected cells, serum ID_{50}s were determined as the serum dilution resulting in a 50% RLU reduction compared to virus-infected untreated controls cells by plotting a nonlinear fit-based agonist vs normalized dose response curve with variable slope and least squares fit in GraphPad Prism 7.0 (GraphPad).

Statistical methods

Statistics were conducted in GraphPad Prism 9.0 (GraphPad). Serum samples that did not show 50% inhibition (ID_{50}) at the lowest tested dilution of 10 (lower limit of quantification, LLOQ) were assigned a value 1/2 of the LLOQ (ID_{50} = 5) for plotting graphs and for statistical

analysis. Group comparisons were done by Mann-Whitney test or Kruskal-Wallis test with Dunn's multiple testing correction, as indicated. Correlation between ID_{50}s and vaccine - infection interval was done by Spearman r.

References

1. Kurth F, Roennefarth M, Thibeault C, Corman VM, Müller-Redetzky H, Mittermaier M, et al. Studying the pathophysiology of coronavirus disease 2019: a protocol for the Berlin prospective COVID-19 patient cohort (Pa-COVID-19). Infection. 2020;48:619–26.

2. Gruell H, Vanshylla K, Tober-Lau P, Hillus D, Schommers P, Lehmann C, et al. mRNA booster immunization elicits potent neutralizing serum activity against the SARS-CoV-2 Omicron variant. [Internet]. Nat Med. 2022;•••. 10.1038/s41591-021-01676-0

3. Hillus D, Schwarz T, Tober-Lau P, Vanshylla K, Hastor H, Thibeault C, et al.; EICOV/COVIM Study Group. Safety, reactogenicity, and immunogenicity of homologous and heterologous prime-boost immunisation with ChAdOx1 nCoV-19 and BNT162b2: a prospective cohort study. [Internet]. Lancet Respir Med. 2021;9:1255–65. 10.1016/S2213-2600(21)00357-X

4. Tober-Lau P, Schwarz T, Vanshylla K, Hillus D, Gruell H, Suttorp N, et al.; EICOV/COVIM Study Group. Long-term immunogenicity of BNT162b2 vaccination in older people and younger health-care workers. Lancet Respir Med. 2021;9:e104–5.

5. Vanshylla K, Di Cristanziano V, Kleipass F, Dewald F, Schommers P, Gieselmann L, et al. Kinetics and correlates of the neutralizing antibody response to SARS-CoV-2 infection in humans. Cell Host Microbe. 2021;29:917–929.e4.

6. Crawford KHD, Eguia R, Dingens AS, Loes AN, Malone KD, Wolf CR, et al. Protocol and Reagents for Pseudotyping Lentiviral Particles with SARS-CoV-2 Spike Protein for Neutralization Assays. [Internet]. Viruses. 2020;12:E513. 10.3390/v12050513

Appendix Table. Characteristics of participants in study of cross-variant neutralizing serum activity after SARS-CoV-2 breakthrough infections

Non-Omicron breakthrough infection cohort, n = 20	Value
Gender, no. (%)	
Male	7 (35)
Female	13 (65)
Age, median years (IQR; range)	39 (28–52; 21–93)
Reported comorbidities, no. (%)	
Arterial hypertension	4 (20)
Asthma	3 (15)
Arrhythmia	3 (15)
Tumor	1 (5)
Diabetes	1 (5)
Sarcoidosis	1 (5)
Time period of SARS-CoV-2 infection	February - November 2021
COVID-19 severity, no. (%)	
Asymptomatic	2 (10)
Mild symptoms	18 (90)
Vaccination received, no. (%)- n (%)	
2 doses BNT162b2	18 (90)
1 dose ChAdOx1 nCoV-19, 1 dose BNT162b2	2 (10)
Sampling time point, median weeks from positive PCR (IQR; range)	6.3 (5.2–7.3; 4.4–9.9)
Time between first and second dose, median weeks (IQR; range)	3.3 (3.0–5.8; 3.0–12.9)
Time between second dose and infection, median weeks (IQR; range)	13.8 (5.1–24.4; 0.6–32.3)
Omicron Breakthrough infection cohort, n = 10	
Gender, no. (%)	
Male	4 (40)
Female	6 (60)
Age, median years (IQR; range)	38 (30-47; 26-56)
Reported comorbidities, no. (%)	
Thyroidectomy	1 (10)
Time period of SARS-CoV-2 infection	December 2021
COVID-19 severity, no. (%)	
Asymptomatic	1 (10)
Mild symptoms	9 (90)
Vaccination received, no. (%)	
2 doses BNT162b2	7 (70)
1 dose ChAdOx1 nCoV-19, 1 dose BNT162b2	2 (20)
2 doses mRNA-1273	1 (10)
Sampling time point, median weeks from positive PCR (IQR; range)	2.1 (2.0-2.3; 2.0-5.7)
Time between first and second dose, median weeks (IQR; range)	6.0 (4.8-6.8; 4.0-10.4)
Time between second dose and infection, median weeks (IQR; range)	22.2 (17.7-24.9; 17.6-30.7)
Vaccinated cohort (*2*), n = 20	
Gender, no. (%)	
Male	7 (35)
Female	13 (65)
Age, median years (IQR, range)	40 (30-54; 27-78)
Reported comorbidities, no. (%)	
Cardiovascular disease	7 (35)
Respiratory disease	3 (15)
Rheumatoid arthritis	1 (5)
Polymyalgia rheumatica	1 (5)
Body mass index, median (IQR; range)	25.6 (22.6-29.8; 18.8-37.0)
Vaccination received	BNT162b2
Sampling time point, median weeks (IQR; range)	
After second dose	3.9 (3.7-4.3; 3.6-6.0)
After third dose	3.3 (3.1-4.1; 2.9-5)
Vaccination intervals, median weeks (IQR; range)	
Time between first and second dose	3.0 (3.0-3.0; 3.0-4.0)
Time between second and third dose	36.7 (33.9-38.8; 26.9-40.9)

*IQR, interquartile range; SARS-CoV-2, severe acute respiratory syndrome coronavirus 2.

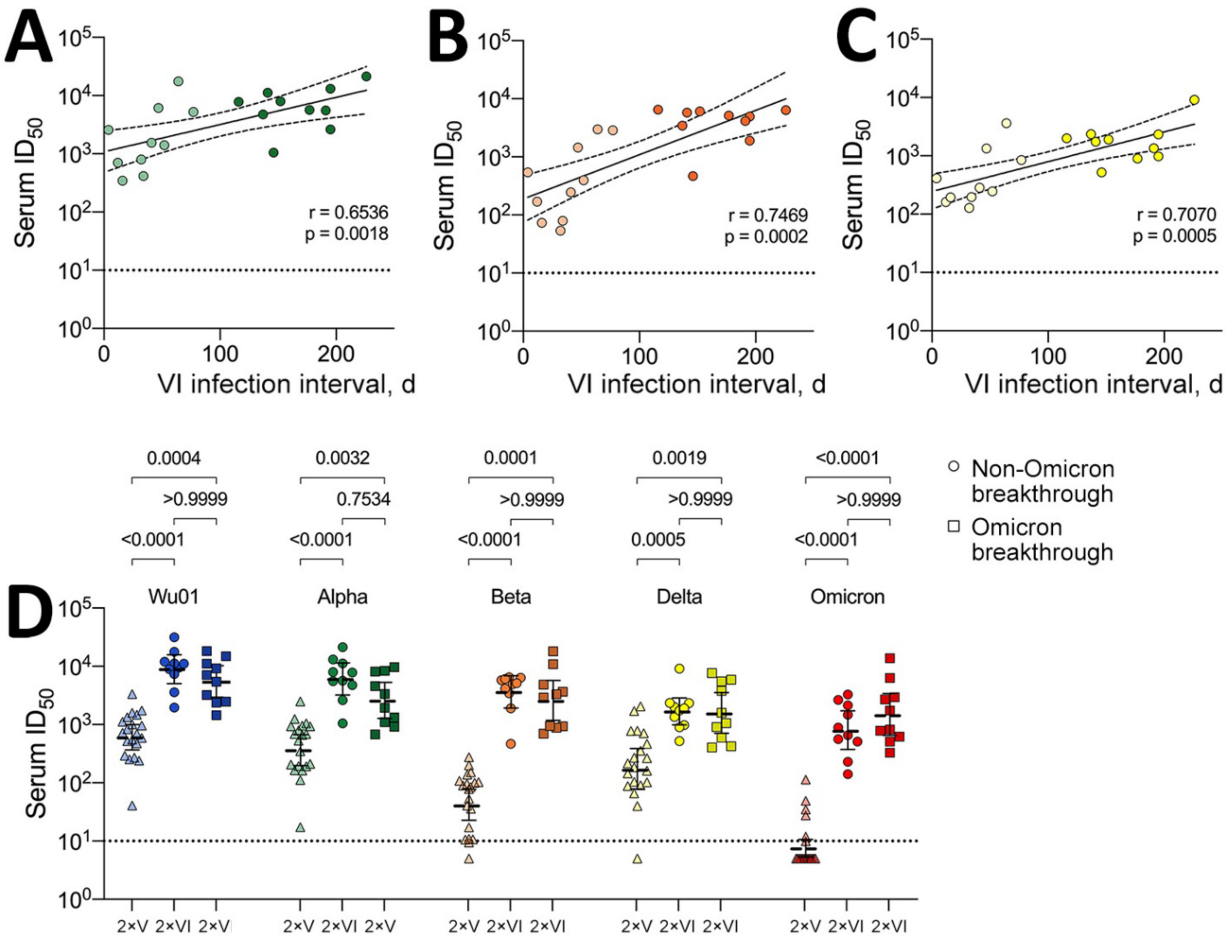

Appendix Figure. Influence of time between vaccination and infection, and infecting SARS-CoV-2 variant on serum neutralizing capacity. A, B, C) Correlation between serum neutralizing activity and interval between second vaccination and non-Omicron breakthrough infection against SARS-CoV-2 Alpha (B, green), Beta (C, orange), and Delta (D, yellow) variants. Breakthrough infections within 3 months (90 days) from vaccination are indicated by light shaded symbols. Lines indicate linear regression with 95% CIs. Correlation was determined by Spearman r. D) Serum neutralizing activity against the indicated SARS-CoV-2 variants in individuals after 2-dose vaccination (triangles), 2-dose vaccination with subsequent non-Omicron (circles) or Omicron (squares) breakthrough infection. Breakthrough infections with vaccine-infection intervals ≥three months are shown. Bars indicate geometric mean ID_{50}s and 95% confidence intervals. Group comparisons by Kruskal-Wallis test. ID_{50}: 50% inhibitory serum dilution; 2x/3x vac: double/triple vaccination; vac-inf: double vaccination with subsequent breakthrough infection; Wu01: SARS-CoV-2 wildtype.
